# Supplementary material for: Expanding the Bacterial Diversity of the Female Urinary Microbiome: Description of Eight New Corynebacterium Species
Source: Microorganisms. 2023 Feb 3;11(2):388. doi: 10.3390/microorganisms11020388 (PMC9963754; doi:10.3390/microorganisms11020388)
Supplement: Supplementary file 1 [file microorganisms-11-00388-s001.zip › microorganisms-2186110-supplementary.pdf]

Expanding the Bacterial Diversity of the Female Urinary Microbiome: Description of Eight New *Corynebacterium* Species

Elisabete Alves Cappelli[1,2], Magdalena Ksiezarek[1,2], Jacqueline Wolf[3], Meina Neumann-Schaal[3], Teresa Gonçalves Ribeiro[1,2,4*] and Luísa Peixe[1,2,4*]

[1] Associate Laboratory i4HB – Institute for Health and Bioeconomy, Faculty of Pharmacy, University of Porto, 4050-313 Porto, Portugal; b.cappelli@hotmail.com (E.A.C); mag.ksiezarek@gmail.com (M.C.); teresampg84@gmail.com (T.G.R.); lpeixe@ff.up.pt (L.P.)

[2] UCIBIO – Applied Molecular Biosciences Unit, Department of Biological Sciences, Faculty of Pharmacy, University of Porto 4050-313 Porto, Portugal; b.cappelli@hotmail.com (E.A.C); mag.ksiezarek@gmail.com (M.C.); teresampg84@gmail.com (T.G.R.); lpeixe@ff.up.pt (L.P.)

[3] Leibniz Institute DSMZ – German Collection of Microorganisms and Cell Cultures GmbH, 38124 Braunschweig, Germany; jacqueline.wolf@dsmz.de (J.W.); meina.neumann-schaal@dsmz.de (M.N.-S.)

[4] CCP – Culture Collection of Porto - Faculty of Pharmacy, University of Porto, 4050-313 Porto, Portugal; teresampg84@gmail.com (T.G.R.); lpeixe@ff.up.pt (L.P.)

* Correspondence: teresampg84@gmail.com (T.G.R.), lpeixe@ff.up.pt (L.P.)

Table S1. Average nucleotide identity between the strains analysed in this study and 46 publicly available genomes of *Corynebacterium*.

Strain	GenBank name	GenBank Accession numbers	Host	Source	8Ua_144	8Ua_174	9Ua_112	19Ua_109	19Ua_121	21Ua68
Marseille-Q3615	*Corynebacterium haemomassiliense*	JACDTZ010000001.1	*Homo sapiens*	Organic fluid (blood)	96.1					
Cj30952	*Corynebacterium jeikeium*	JFCR01000001.1	*Homo sapiens*	Vertebral disk space		97.1				
HMSC08A12	*Corynebacterium* sp.	LWNW01000001.1	*Homo sapiens*	Urine		97.0				
Cj38002_200	*Corynebacterium jeikeium*	JFCP01000001.1	*Homo sapiens*	Unknown			97.6			
Cj19409	*Corynebacterium jeikeium*	JFCI01000001.1	*Homo sapiens*	Spine			97.5			
Cj37130	*Corynebacterium jeikeium*	JFCQ01000001.1	*Homo sapiens*	Knee bursa			97.4			
Cj47453 261	*Corynebacterium jeikeium*	JFCL01000001.1	*Homo sapiens*	Pleural fluid			96.8			
Cj14566 186	*Corynebacterium jeikeium*	JFCF01000001.1	*Homo sapiens*	Unknown			97.4			
Cj30184 2468	*Corynebacterium jeikeium*	JFCK01000001.1	*Homo sapiens*	Unknown			97.5			
Cj21382 127	*Corynebacterium jeikeium*	JFCJ01000001.1	*Homo sapiens*	Humeral membrane			97.4			
Cj16348 216	*Corynebacterium jeikeium*	JFCG01000001.1	*Homo sapiens*	Unknown			97.3			
HMSC058E07	*Corynebacterium* sp.	LTIW01000001.1	*Homo sapiens*	Tissue			97.5			
LK28	*Corynebacterium* sp.	PDGZ01000001.1	*Homo sapiens*	Diabetic foot ulcer			97.4			
Cj47446	*Corynebacterium jeikeium*	JFCN01000001.1	*Homo sapiens*	Decubitus ulcer			97.2			
HMSC068H04	*Corynebacterium* sp.	LTGK01000001.1	*Homo sapiens*	Vaginal/Rectal				96.8		
DE0414	*Corynebacterium aurimucosum*	VDSY01000001.1	Not applicable	Environmental				96.8		
2299	*Corynebacterium aurimucosum*	VKDI01000001.1	*Homo sapiens*	Urine				96.6		
DE0410	*Corynebacterium aurimucosum*	VDTC01000001.1	Not applicable	Environmental				96.8		

DE0412	*Corynebacterium aurimucosum*	VDTA01000001.1	Not applicable	Environmental				96.8		
DE0409	*Corynebacterium aurimucosum*	VDTD01000001.1	Not applicable	Environmental				96.8		
DE0142	*Corynebacterium aurimucosum*	VEFK01000001.1	Not applicable	Environmental				96.8		
DE0407	*Corynebacterium aurimucosum*	VDTF01000001.1	Not applicable	Environmental				96.8		
DE0415	*Corynebacterium aurimucosum*	VDSX01000001.1	Not applicable	Environmental				96.8		
DE0416	*Corynebacterium aurimucosum*	VDSW01000001.1	Not applicable	Environmental				96.8		
DE0417	*Corynebacterium aurimucosum*	VDSV01000001.1	Not applicable	Environmental				96.8		
DE0418	*Corynebacterium aurimucosum*	VDSU01000001.1	Not applicable	Environmental				96.8		
2271	*Corynebacterium aurimucosum*	VKDG01000001.1	*Homo sapiens*	Urine				96.5		
2218	*Corynebacterium aurimucosum*	VKDH01000001.1	*Homo sapiens*	Urine				96.6		
2226	*Corynebacterium aurimucosum*	VKDJ01000001.1	*Homo sapiens*	Urine				96.6		
13	*Corynebacterium aurimucosum*	VIOF01000001.1	*Homo sapiens*	Sebaceous cyst				96.4		
HMSC055A01	*Corynebacterium* sp.	LTJU01000001.1	*Homo sapiens*	Urine				96.4		
HMSC056E09	*Corynebacterium* sp.	LTWQ01000001.1	*Homo sapiens*	Urine				96.5		
HMSC078C09	*Corynebacterium* sp.	LTUR01000001.1	*Homo sapiens*	Urine				96.8		
HMSC072A02	*Corynebacterium* sp.	LTIM01000001.1	*Homo sapiens*	Urine				96.5		
HMSC059E07	*Corynebacterium* sp.	LTUY01000001.1	*Homo sapiens*	Urine				96.3		
HMSC062E11	*Corynebacterium* sp.	LTFH01000001.1	*Homo sapiens*	Vaginal/Rectal				96.5		
HMSC065D07	*Corynebacterium* sp.	LTHJ01000001.1	*Homo sapiens*	Urine				96.5		
ACRQA	*Corynebacterium aurimucosum*	JAKRDE010000001.1	*Homo sapiens*	Skin				96.3		

HESMS017b	*Corynebacterium aurimucosum*	JADCSP010000001.1	*Homo sapiens*	Nasal mucosa				96.0		
UMB7769	*Corynebacterium aurimucosum*	JAAUWA010000001.1	*Homo sapiens*	Urine				96.4		
UMB0043	*Corynebacterium aurimucosum*	PKHR01000001.1	*Homo sapiens*	Urine				96.6		
620 CAUR	*Corynebacterium aurimucosum*	JUZU01000001.1	*Homo sapiens*	Unknown					96.0	
FDAARGOS 993	*Corynebacterium tuberculostearicum*	CP065972.1	Unknown	Unknown					95.9	
ACRPP	*Corynebacterium aurimucosum*	JAKRDY010000001.1	*Homo sapiens*	Skin					96.6	
911 CAUR	*Corynebacterium aurimucosum*	JUOH01000001.1	*Homo sapiens*	Unknown					95.9	
BWA136	*Corynebacterium* sp.	JAKOPM010000001.1	*Homo sapiens*	Nasopharynx						99.9

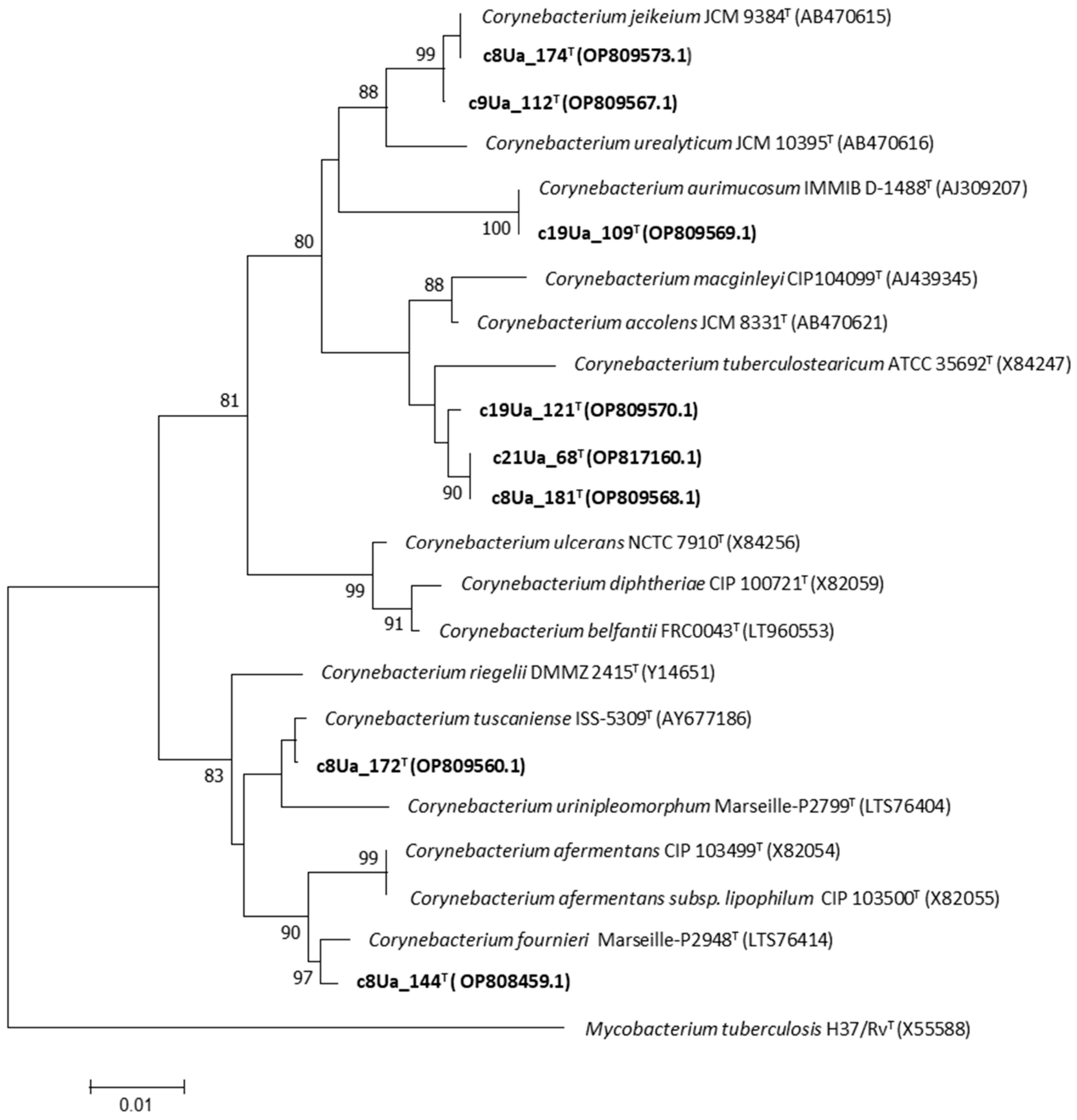

Fig. S1. Neighbour-joining tree (Kimura's two-parameter model and pairwise-deletion option) based on 16S rRNA gene sequences showing the phylogenetic relationships of the strains c8Ua_144^{T}, c8Ua_172^{T}, c8Ua_174^{T}, c8Ua_181^{T}, c9Ua_112^{T}, c19Ua_109^{T}, c19Ua_121^{T}, c21Ua_68^{T}, and closely related type strains of the genus *Corynebacterium*. *Mycobacterium tuberculosis* H37Rv was used as the outgroup. Bootstrap percentages (based on 1000 replications) are shown at nodes. Only values above 80% are shown. Bar, 0.01 substitutions per nucleotide position.

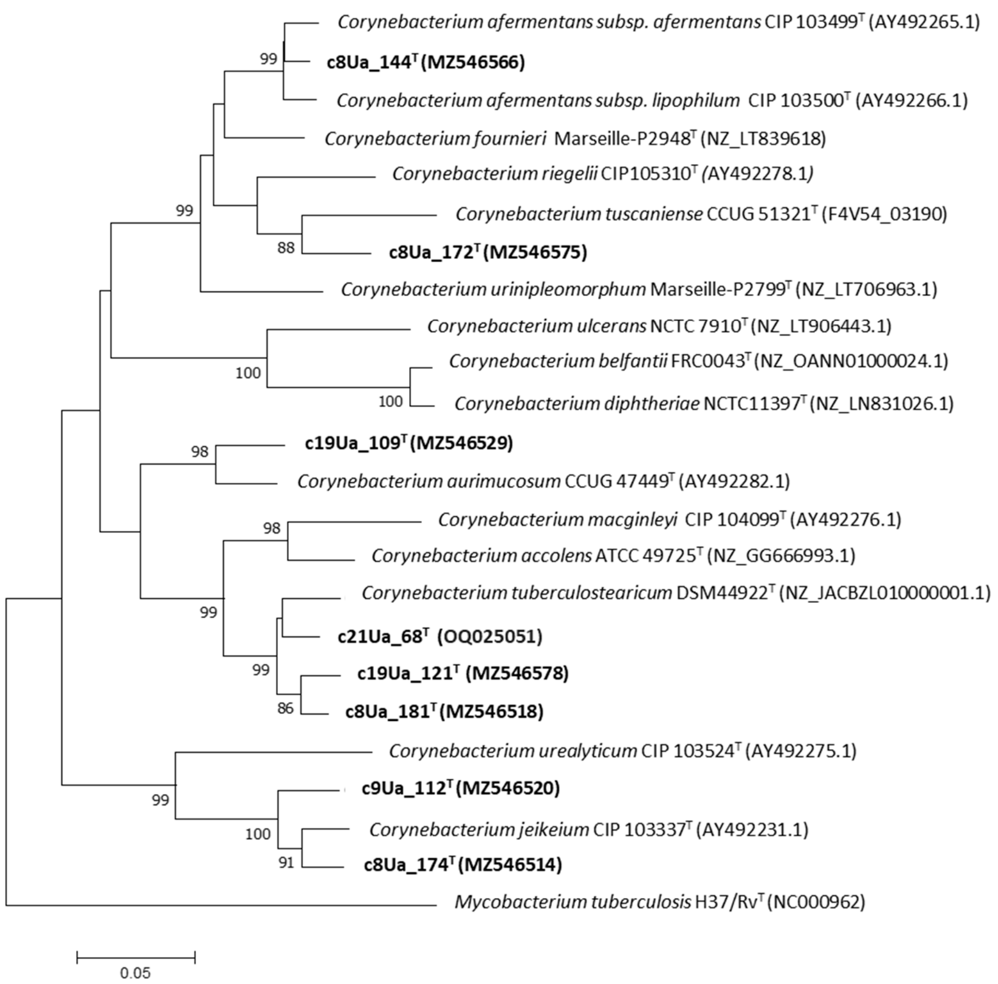

Fig. S2. Neighbour-joining tree (Kimura's two-parameter model and pairwise-deletion option) based on *rpoB* gene sequences showing the phylogenetic relationships of *Corynebacterium* c8Ua_144^T, c8Ua_172^T, c8Ua_174^T, c8Ua_181^T, c9Ua_112^T, c19Ua_109^T, c19Ua_121^T, c21Ua_68^T, and closely related type strains of the genus *Corynebacterium*. *Mycobacterium tuberculosis* H37Rv was used as the outgroup. Bootstrap percentages (based on 1000 replications) are shown at nodes. Only values above 80% are shown. Bar, 0.05 substitutions per nucleotide position.

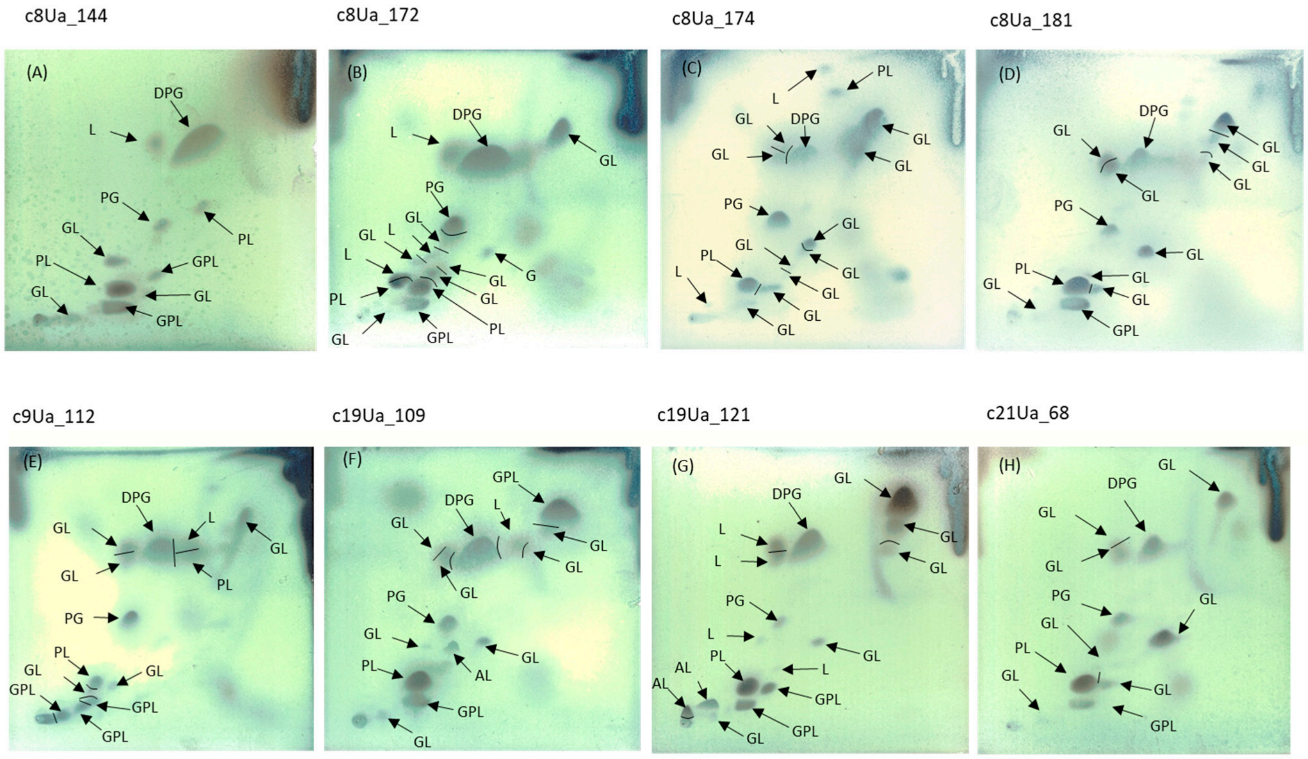

Fig. S3. Two-dimensional thin layer chromatogram (TLC) of extracted lipids of: c8Ua_144^T (A), c8Ua_172^T (B), c8Ua_174^T (C), c8Ua_181^T (D), c9Ua_112^T (E), c19Ua_109^T (F), c19Ua_121^T (G), c21Ua_68^T (H). AL, aminolipids; DPG, diphosphatidylglycerol; GL, glycolipids; GPL, glycophospholipids; L, lipids; PG, phophatidylglycerol; PL, Phospholipids.
